# Homeopathy in Veterinary Practice1Read before the New Hampshire Veterinary Medical Association, May 5, 1896.

**Published:** 1896-07

**Authors:** A. V. Abbott


### HOMEOPATHY IN VETERINARY PRACTICE.[1]

By A. V. ABBOTT, D.V.S.

In presenting this paper for the consideration and discussion of this Society to-day I am fully aware that I shall be obliged to defend it alone, as I think none of you practice by this method. Such being the case, coupled with my limited knowledge of the subject, I anticipate in your judgment "coming out at the small end of the horn," as is the common expression, but I shall nevertheless endeavor to bring some of its advantages as seen by me to your attention.

First of all, what is homeopathy? You have already heard

[1] Read before the New Hampshire Veterinary Medical Association, May 5, 1896.

the doctrine, *similia similibus curantur*, until you know perfectly well what it means, so I will pass that by. Another has called it nature's law of healing. We have all, no doubt, thought to ourselves how little we could do with our medicine unless nature should be willing to help us. That leads us, then, to think that we must try to help and not force nature. Nature never purges the bowels unless there is some obnoxious material contained therein or other abnormal cause acting. Well, then, you say, if we give a small dose of aloes or oil in this case, are we not helping nature to throw out this material? Certainly, and if the aloes, oil, or other drug is selected with regard to its homeopathic relation and given in a small dose, it will check it and not leave as bad a case of constipation as we formerly had of purgation, either. Of course, some of the old ideas and teachings as regards minor points are not in vogue to-day, but still the grand foundation of the work remains the same, and will as long as it is called homeopathy. Surgery, engineering, electricity, chemistry, and, in fact, all the sciences and arts, have made great progress within the last few years, and you surely would not expect homeopathy to stand still.

Now then, if nature is to help us, and we in turn try to help nature, tell me the reason in giving medicine in such powerful doses as will cause its effects to be felt and nature to be disturbed and perverted for long periods after the disease has disappeared. Surely this is not desirable. We have often heard people remark that their eyes have been weak ever since they were sick with this disease, or that they have had heart-trouble ever since they had that once, and similar remarks. We cannot say it is always the case, but still quite frequently, that these effects can be accounted for if we look at it from a homeopathic standpoint, and occasionally you will hear the remark from an allopathic source. In the late war, I have been told by a soldier, it was the custom, when a man felt a little indisposed, to get a big dose of quinine and wash it down with a stimulant. He said it made no difference what the trouble was, especially at the start, they always took that first. The same man, who was not a homeopathist, also said the effects were still left, and he expected to carry them to his grave.

Probably a few of the more important points, as well as some of the fundamental rules, would be of interest in helping us to understand true homeopathy. The homeopathist explains his theory something like this : If a certain symptom or a collection of symptoms is seen in a case, there is a certain nerve-centre or set of nerve-centres affected by something not found in the healthy body. Now, if a certain drug in any healthy body will cause these same symptoms, it is reasoned that the same set of centres are affected by the drug. This is certainly a rational way of explaining it. Then if this same drug is given in a very small quantity it will act on and correct or modify the action of the same centres. The fundamental principles as given by Dewey are these:

1. That disease is manifested by symptoms.

2. That knowledge of drug-action should be obtained by experimentation on a healthy body.

3. That the curative relation between these two sets of phenomena is the law of similars, or *similia similibus curantur.*

4. That the selected remedy should be given singly and uncombined with any other.

5. That it should be given in the smallest dose that will effect a cure.

The only indication needed for the selection of a remedy is the totality of the symptoms in the particular case. In this totality even the symptoms are not all of like importance, for the more prominent, uncommon, and peculiar need the most attention, for often the general symptoms are common to more than one drug. These peculiar symptoms of any disease are termed characteristic symptoms, and are always the safest to follow in the selection of a drug. In fact, I have often had good results in prescribing where I have done so on one symptom alone, providing that stood out prominently. We sometimes get an aggravation after a varying period of administration, as often is the case with phosphorus, which is looked upon as a favorable sign, and proof that the remedy selected has been the correct one.

Absolute cleanliness should be ever sought for in preparing and dispensing medicines. A bottle once used for medicine is unfit for another kind or higher attenuations of the same kind ; but I am satisfied if it has been washed out in boiling water, and contains no odor, when using the mother-tincture or the first potency.

The medicines used are either in the form of tablets, triturations, or dilutions. A trituration is a medicine combined and thoroughly mixed with sugar of milk which has been recrystallized. Tablets are a compressed form of the trituration. A dilution is the medicine mixed with alcohol. I use water usually on account of the price, and fail to see why alcohol is better, providing the water is pure, unless it is to be kept for some time. A strong tincture, one-sixth drug strength usually, is called a mother-tincture. The IX. is one part of the tincture and nine of alcohol. The 2X. is one part of the IX. and nine of alcohol, and so on. The triturations are made with the salts of metals and minerals, rubbed up with sugar of milk, and in the same proportions. Many of the remedies, such as sulphur, lycopodium, psorinum, etc., are claimed to act far better in the higher potencies, as, for instance, the thirtieth. Some think all the remedies act better high and others low, and I myself find that a good deal depends on the drug used; but all this is of minor importance and should be left to one's own ideas and experience, for the question in homeopathy is not the dose, but the remedy.

Some of its advantages over the old school may be enumerated as follows: It cures disease in the safest and quickest manner. Dangerous drugging and debilitating measures are no part of its teachings. Its cost is certainly advantageous. Diseases beyond the reach of allopathic medication are cured by it. Its use often renders the knife unnecessary in the treatment of tumors, etc. The development of diseases and malignant growths are prevented in their incipiency. And when we come to its administration, see how much more convenient it is for your client or yourself to place a few drops of a solution or a small powder on the tongue of your patient than it is to use a syringe, give a pill, or pour a drench into a horse which heartily objects to the bottle. Of course, its cost and administration are of secondary importance, providing the effects of the treatment should be favorable to the other system; but if they are not, these two should certainly be considered. I fully believe in homeopathy, although I do not as yet use it in the treatment of all diseases, and for this reason: There are a few diseases which yield very satisfactorily to allopathic treatment, and, in fact, it would be hard to improve upon their treatment, except as a matter of cost or administration, so I have delayed their homeopathic treatment until such a time as I can have studied enough to be thoroughly acquainted with the materia medica.

I suppose the most interesting part of this paper will be the account of its application in a few cases which have come under my notice, and I hope you will not connect anything that is said with self-conceit, but rather place it where it is intended to go, viz., to the credit of homeopathy. The first two or three cases will show its effects upon injuries of the tendons and muscles. Was asked to look at a brown gelding, five years of age, which had been castrated in the early spring of 1895, and in the process of casting and the operation his loins had been severely sprained, and at the time I first saw him, some four or five months after the operation, he came out of the box on his toes, with the heels of both hindfeet raised from the floor about three-quarters of an inch. There was also considerable soreness evinced by slight pressure on the lumbar muscles. I left a small bottle of rhus tox. 2X., enough to go about ten days, with directions that he should receive fifteen drops four times a day, and that he should be exercised as usual, which was an hour's driving each day, but to avoid tiring. My client was to report to me when the medicine was gone, at which time I would either change or repeat it. Not hearing from the man as agreed, I hunted him up one day, when in answer to my query as to his negligence in not reporting to me before, he replied that he thought it unnecessary, as his colt was all right, and he had some of the medicine left.

One Tuesday was called to see a roan gelding, which was very lame on the near front, and suffering from strained tendons and suspensory ligament. The tendons were swollen so that the leg was perfectly round, and it was impossible to extend the limb fully. He was driven the following Sunday after receiving rhus internally and externally. The medicine was continued for a week longer, however.

I have treated a great many cases found in the same condition with like satisfactory results, but some of them required a little more time and patience, although not near the time required for firing and blistering. I have not tried this treatment in any chronic cases of the same kind, principally because the thermo-cautery gives good results both to our clients and to our own pocket-books.

December 8, 1895, was called to see roan filly, two years of age, which had been going lame for a few weeks. Could find no acute lesions, but a little soreness in the external scapula muscles. As the filly was very narrow-chested and rather light in the forequarters generally, I attributed it to a weakness of the muscles, probably aggravated by a slip or fall. She received arnica internally, and the same made into a lotion externally. After three weeks she went sound, and has remained so since. I have found arnica to bear about the same relation to the muscles that rhus does to the fibrous structures.

I have given nothing but the homeopathic remedies in colic for a year now, and have had to record no fatal cases with the exception of one, which revealed a ruptured stomach on post-mortem examination. I have divided the treatment between colocynth, chamomilla, aconite, belladonna, and nux vomica, principally, given according to the symptoms presented. In pneumonia and bronchitis I have discarded all allopathic treatment, relying upon aconite, belladonna, phosphorus, mercurius, bryonia, tartar emetic, gelseminum, arsenicum, lycopodium, and iodine, given as the symptoms called for them. I have had opportunity to try it in but one case of parturient apoplexy, which came out very satisfactorily after being down five days. In all the minor troubles, such as catarrh, laryngitis, pharyngitis, vaginitis, diuresis, leucorrhea, bruises, etc., I use it exclusively. In conjunctivitis and periodic ophthalmia I have had most excellent results.

Now, in discussing its successful results, we must admit one of two points, viz., that the treatment has cured the disease, or that the same would have recovered as satisfactorily without any treatment, which, of course, would be hard to deny, no matter what the treatment. We certainly must honor one of these points.

Many of the allopathic medicines are used in strict accordance with the law of similars, although they are explained in an entirely different manner. Belladonna is a constituent of nearly all laryngeal cough-mixtures and electuaries, and if you will look in a homeopathic materia medica you will find that "the larynx is sore and hot," "great dryness and bright redness of the throat," "the fauces are inflamed, and food and liquids are ejected through the nose on swallowing;" also "that there is a dry, hacking cough" as its principal throat-symptoms. Nux vomica is given frequently in inactivity of the bowels, and, according to Finlay Dunn, "it overcomes chronic constipation." If we look in a homeopathic materia medica we find that "prolonged and severe costiveness with intestinal inertia" is one of its chief symptoms. The recent antitoxin treatment for the various diseases belongs to the system of isopathy, which in some ways resembles homeopathy very much. Finally, I will conclude, gentlemen, by asking a few favors and giving a little advice. Do not rely upon what I have said for the treatment of any disease until you have found the indications as given in some reliable work. Do not try it at all until you have made yourself pretty well acquainted with its principles and have come to the conclusion that it is at least worth trying. Do not be too harsh in your judgment of it until you have given it as reasonable a trial as you would some new agent in the old school. If I am unable to answer some of the questions put to me in regard to it, please remember that I am yet young in its study, and that I have simply intended to open the subject, hoping that thereby you will be induced to inquire into its teachings in your leisure time, and, if found worthy, to give them a good fair trial in your practice.

---

*Coaching*, a Philadelphia paper devoted to the interests of horsemen, has changed its name to *Whip and Spur*.

An Alsatian has arranged a suitable harness with pole-gearing attachment passing from the bicycle to the rear, by which he utilizes his dog as a motor-power for propelling him along on his rides.

Sweden reports a radiator machine by which butter may be made from sterilized milk in one minute.

Wild animals are not exempt from the ills of seasickness when being transported from one country to another, and present many curious groups of symptoms at times.

The *Breeder's Gazette* says Salem County, Missouri, has the largest mule. He stands 21 hands high and weighs 2000 pounds; Ohio the smallest horse, two years old, and only 13 inches high.

A great many of the leading breeders of dogs in our country have already sent valuable contributions to the new kennels at Washington, D. C., and the projectors are highly encouraged at the prospect of having a valuable display at the national "Zoo."

The consulting veterinarian to the Humane Society of Washington, D. C., attaches the handle of homeopathic veterinary physician and surgeon to his name.

